# Cholest-5-ene

**DOI:** 10.1107/S1600536811016783

**Published:** 2011-05-11

**Authors:** Mohd Shaheen Khan, Othman Sulaiman, Rokiah Hashim, Madhukar Hemamalini, Hoong-Kun Fun

**Affiliations:** a Bioresource, Paper and Coatings Technology Division, School of Industrial Technology, Universiti Sains Malaysia, 11800 USM, pulau Penang, Malaysia; b X-ray Crystallography Unit, School of Physics, Universiti Sains Malaysia, 11800 USM, Penang, Malaysia

## Abstract

The asymmetric unit of the title compound, C_27_H_46_, contains two crytallographically independent cholest-5-ene mol­ecules (*A* and *B*). In each mol­ecule, the three six-membered rings are all in chair conformations, while the five-membered ring is in a twist conformation. The terminal isopropyl group of mol­ecule *A* has a (−)-gauche conformation, whereas that of mol­ecule *B* has a (+)-gauche conformation. No significant inter­molecular inter­actions are observed in the crystal structure.

## Related literature

For details of steroidal heterocyclic derivatives, see: Kwon *et al.* (1995 ▶); Jindal *et al.* (2001 ▶); Hoyte *et al.* (2002 ▶). For reported melting-point details, see: Dauben & Takemura (1953 ▶). For a related structure, see: Coles *et al.* (2002 ▶). For ring conformations, see: Cremer & Pople (1975 ▶). For the stability of the temperature controller used in the data collection, see: Cosier & Glazer (1986 ▶). 
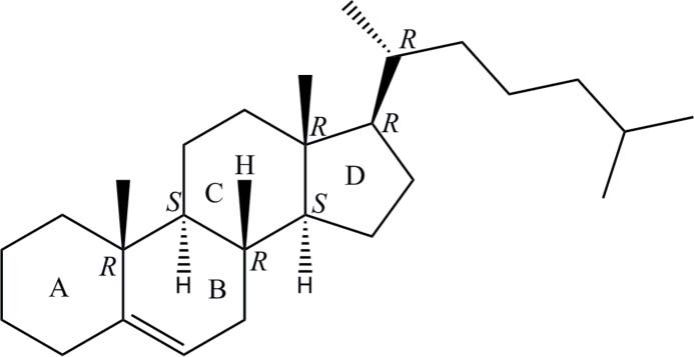


## Experimental

### 

#### Crystal data

C_27_H_46_
*M* *_r_* = 370.64
Monoclinic, 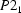

*a* = 10.6938 (2) Å
*b* = 19.4062 (3) Å
*c* = 11.1763 (2) Å
β = 93.663 (1)°
*V* = 2314.64 (7) Å^3^
*Z* = 4
Mo *K*α radiation
μ = 0.06 mm^−1^
*T* = 100 K
0.43 × 0.35 × 0.22 mm

#### Data collection

Bruker SMART APEXII CCD area-detector diffractometer
Absorption correction: multi-scan (*SADABS*; Bruker, 2009 ▶) *T*_min_ = 0.975, *T*_max_ = 0.987
49622 measured reflections
8594 independent reflections
7317 reflections with *I* > 2σ(*I*)
*R*_int_ = 0.046

#### Refinement

*R*[*F* ^2^ > 2σ(*F* ^2^)] = 0.054
*wR*(*F* ^2^) = 0.124
*S* = 1.05
8594 reflections
497 parameters
1 restraint
H-atom parameters constrained
Δρ_max_ = 0.33 e Å^−3^
Δρ_min_ = −0.23 e Å^−3^

### 

Data collection: *APEX2* (Bruker, 2009 ▶); cell refinement: *SAINT* (Bruker, 2009 ▶); data reduction: *SAINT*; program(s) used to solve structure: *SHELXTL* (Sheldrick, 2008 ▶); program(s) used to refine structure: *SHELXTL*; molecular graphics: *SHELXTL*; software used to prepare material for publication: *SHELXTL* and *PLATON* (Spek, 2009 ▶).

## Supplementary Material

Crystal structure: contains datablocks global, I. DOI: 10.1107/S1600536811016783/rz2593sup1.cif

Structure factors: contains datablocks I. DOI: 10.1107/S1600536811016783/rz2593Isup2.hkl

Additional supplementary materials:  crystallographic information; 3D view; checkCIF report

## Figures and Tables

**Fig. 1.**
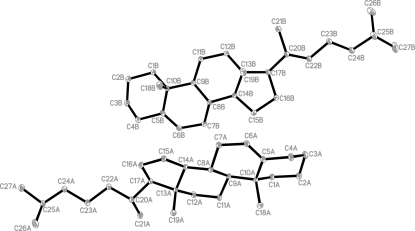
The asymmetric unit of the title compound. Displacement ellipsoids are drawn at the 30% probability level. H atoms are omitted for clarity.

## supplementary crystallographic information

### Comment

A literature survey reveals that considerable attention has been devoted to the synthesis of several steroidal heterocyclic derivatives that exhibit marked medicinal activities (Kwon et al., 1995; Jindal et al., 2001; Hoyte et al., 2002). Steroidal derivatives have been found to possess a variety of interesting pharmacological and biological activities. In view of the biological importance  of cholesterols, we report here the crystal structure of a new cholesterol derivative.

Cholest-5-ene contains two molecules (A and B) in the asymmetric unit, as shown in Fig 1. The bond lengths and angles agree well with those of other cholesterol derivatives. The conformations are quite similar for the tetracyclic ring systems in molecules A and B. The structure is composed of four fused carbon rings with two methyl substituents, and an alkyl side chain. In each molecule, the three six-membered rings are all in the chair conformation [Ring A (C1A–C5A/C10A):(C1B–C5B/C10B): Q = 0.540 (2)/0.554 (2) Å; θ = 4.1 (2)/9.7 (2)° and φ = 80 (3)/107.0 (13)°.  Ring B (C5A–C10A):(C5B–C10B): Q = 0.4731 (19)/0.4906 (19) Å; θ = 51.7 (2)/50.8 (2)° and φ= 208.2 (3)/209.4 (3)° and Ring C (C8A–C9A/(C11A–C14A): (C8B–C9B/(C11B–C14B): Q = 0.5709 (19)/0.5631 (19) Å; θ = 7.14 (19)/5.31 (19)° and φ = 262.6 (15)/229.0 (2)°], whilst, according to puckering analysis (Cremer & Pople, 1975), the five-membered ring is in a twist conformation [D (C13A–C17A):(C13B–C17B): Q = 0.4476 (19)/0.4824 (19)Å and θ = 194.5 (2)/185.7 (2)°].

The crystal structure of 5α-cholestane has been previously reported by Coles et al. (2002). The present structure is very similar to the previously reported 5α-cholestane [C5–C6 = 1.518 (4) Å in molecule A and 1.523 (4) Å in molecule B].  Here, we observed that the C5–C6 bond length is 1.331 (2) Å in molecule A and 1.331 (3) Å in molecule B, clearly indicating that C5═C6 is a double bond.

The C17–C27 tails of the two molecules are almost fully extended, as in most cholesterol derivatives. The torsion angles C23A-C24A-C25A-C26A of 59.8 (2)° and C23A-C24A-C25A-C27A of -175.69 (18)° show that the terminal isopropyl group has a (-)-gauche conformation in molecule A, whereas molecule B has a (+)-gauche conformation [C23B-C24B-C25B-C27B = 173.0 (2)° and C23B-C24B-C25B-C26B = -62.6 (3)°].

There are seven chiral centres in each molecule. From the structure presented, these centers exhibit the following relative chiralities: C8A = R; C9A = S; C10A = R; C13A = R; C14A = S; C17A = R and C20A = R (molecule A), C8B = R; C9B = S; C10B = R; C13B = R; C14B = S; C17B = R and C20B = R (molecule B). The absolute configuration cannot be determined as there is not enough anomalous dispersion.

In the crystal structure, no significant intermolecular interactions are observed.

### Experimental

3β-Chlorocholest-5-ene (10 g) was dissolved in warm amyl alcohol (230 mL) and sodium metal (20 g) was added in small portions to the solution with continuous stirring over a period of 8 h. The reaction mixture was warmed occasionally. When all sodium metal was dissolved, the reaction mixture was poured into water, acidified with dilute hydrochloric acid and allowed to stand overnight. A white crystalline solid obtained was filtered under suction and washed thoroughly with water and air dried. Recrystallization of the crude material from acetone yielded colourless cubic-shaped cholest-5-ene crystals (8.3 g). M.p. 93°C (reported m.p. 89–91.2°C; Dauben & Takemura, 1953).

### Refinement

All hydrogen atoms were positioned geometrically [C–H = 0.98–1.00 Å] and were refined using a riding model, with *U*_iso_(H) = 1.2 *U*_eq_(C) or 1.5 *U*_eq_(C) for methyl H atoms. A rotating group model was applied to the methyl groups. 7194 Friedel pairs were merged for the final refinement as there is not enough anomalous dispersion to determine the absolute configuration.

### Crystal data

**Table:**

C_27_H_46_	*F*(000) = 832
*M**_r_* = 370.64	*D*_x_ = 1.064 Mg m^−^^3^
Monoclinic, *P*2_1_	Mo *K*α radiation, λ = 0.71073 Å
Hall symbol: P 2yb	Cell parameters from 9842 reflections
*a* = 10.6938 (2) Å	θ = 2.8–32.4°
*b* = 19.4062 (3) Å	µ = 0.06 mm^−^^1^
*c* = 11.1763 (2) Å	*T* = 100 K
β = 93.663 (1)°	Block, colourless
*V* = 2314.64 (7) Å^3^	0.43 × 0.35 × 0.22 mm
*Z* = 4	

### Data collection

**Table:**

Bruker SMART APEXII CCD area-detector diffractometer	8594 independent reflections
Radiation source: fine-focus sealed tube	7317 reflections with *I* > 2σ(*I*)
graphite	*R*_int_ = 0.046
φ and ω scans	θ_max_ = 32.6°, θ_min_ = 1.9°
Absorption correction: multi-scan (*SADABS*; Bruker, 2009)	*h* = −15→16
*T*_min_ = 0.975, *T*_max_ = 0.987	*k* = −29→28
49622 measured reflections	*l* = −16→15

### Refinement

**Table:**

Refinement on *F*^2^	Primary atom site location: structure-invariant direct methods
Least-squares matrix: full	Secondary atom site location: difference Fourier map
*R*[*F*^2^ > 2σ(*F*^2^)] = 0.054	Hydrogen site location: inferred from neighbouring sites
*wR*(*F*^2^) = 0.124	H-atom parameters constrained
*S* = 1.05	*w* = 1/[σ^2^(*F*_o_^2^) + (0.0666*P*)^2^ + 0.2376*P*] where *P* = (*F*_o_^2^ + 2*F*_c_^2^)/3
8594 reflections	(Δ/σ)_max_ = 0.001
497 parameters	Δρ_max_ = 0.33 e Å^−^^3^
1 restraint	Δρ_min_ = −0.23 e Å^−^^3^

### Special details

**Table:**

Experimental. The crystal was placed in the cold stream of an Oxford Cryosystems Cobra open-flow nitrogen cryostat (Cosier & Glazer, 1986) operating at 100.0 (1) K.
Geometry. All s.u.'s (except the s.u. in the dihedral angle between two l.s. planes) are estimated using the full covariance matrix. The cell s.u.'s are taken into account individually in the estimation of s.u.'s in distances, angles and torsion angles; correlations between s.u.'s in cell parameters are only used when they are defined by crystal symmetry. An approximate (isotropic) treatment of cell s.u.'s is used for estimating s.u.'s involving l.s. planes.
Refinement. Refinement of F^2^ against ALL reflections. The weighted R-factor wR and goodness of fit S are based on F^2^, conventional R-factors R are based on F, with F set to zero for negative F^2^. The threshold expression of F^2^ > 2σ(F^2^) is used only for calculating R-factors(gt) etc. and is not relevant to the choice of reflections for refinement. R-factors based on F^2^ are statistically about twice as large as those based on F, and R- factors based on ALL data will be even larger.

### Fractional atomic coordinates and isotropic or equivalent isotropic displacement parameters (Å^2^)

**Table:**

	*x*	*y*	*z*	*U*_iso_*/*U*_eq_	
C13A	0.37488 (16)	0.40754 (9)	0.78901 (14)	0.0156 (3)	
C12A	0.27174 (17)	0.36278 (9)	0.83954 (16)	0.0188 (3)	
H12A	0.2000	0.3595	0.7793	0.023*	
H12B	0.2419	0.3851	0.9120	0.023*	
C11A	0.31885 (18)	0.28969 (9)	0.87192 (15)	0.0189 (3)	
H11A	0.2476	0.2621	0.8981	0.023*	
H11B	0.3818	0.2929	0.9406	0.023*	
C9A	0.37803 (17)	0.25134 (9)	0.76830 (14)	0.0160 (3)	
H9AA	0.3089	0.2440	0.7050	0.019*	
C10A	0.42859 (17)	0.17816 (9)	0.80370 (14)	0.0161 (3)	
C1A	0.31619 (18)	0.12919 (9)	0.81767 (16)	0.0193 (3)	
H1AA	0.2719	0.1438	0.8885	0.023*	
H1AB	0.2570	0.1341	0.7464	0.023*	
C2A	0.3517 (2)	0.05301 (10)	0.83210 (17)	0.0233 (4)	
H2AA	0.4029	0.0465	0.9082	0.028*	
H2AB	0.2747	0.0250	0.8358	0.028*	
C3A	0.4255 (2)	0.02846 (10)	0.72775 (18)	0.0265 (4)	
H3AA	0.4524	−0.0199	0.7417	0.032*	
H3AB	0.3712	0.0300	0.6526	0.032*	
C4A	0.5399 (2)	0.07375 (10)	0.71500 (18)	0.0247 (4)	
H4AA	0.5995	0.0668	0.7855	0.030*	
H4AB	0.5823	0.0595	0.6427	0.030*	
C5A	0.50649 (17)	0.14922 (9)	0.70493 (15)	0.0186 (3)	
C6A	0.54370 (18)	0.18658 (9)	0.61387 (15)	0.0199 (3)	
H6AA	0.5885	0.1636	0.5549	0.024*	
C7A	0.52013 (18)	0.26199 (9)	0.59759 (15)	0.0194 (3)	
H7AA	0.4556	0.2688	0.5311	0.023*	
H7AB	0.5981	0.2846	0.5748	0.023*	
C8A	0.47629 (17)	0.29661 (9)	0.71085 (14)	0.0164 (3)	
H8AA	0.5500	0.3026	0.7696	0.020*	
C14A	0.42058 (16)	0.36728 (9)	0.68059 (14)	0.0158 (3)	
H14A	0.3444	0.3587	0.6260	0.019*	
C15A	0.50026 (18)	0.41878 (9)	0.61573 (16)	0.0195 (3)	
H15A	0.5034	0.4067	0.5299	0.023*	
H15B	0.5868	0.4206	0.6528	0.023*	
C16A	0.43156 (18)	0.48791 (9)	0.63127 (15)	0.0199 (3)	
H16A	0.3898	0.5028	0.5540	0.024*	
H16B	0.4919	0.5241	0.6592	0.024*	
C17A	0.33231 (17)	0.47615 (9)	0.72619 (15)	0.0162 (3)	
H17A	0.2501	0.4670	0.6812	0.019*	
C20A	0.31612 (17)	0.54145 (9)	0.80253 (15)	0.0180 (3)	
H20A	0.3994	0.5528	0.8435	0.022*	
C22A	0.27633 (19)	0.60253 (9)	0.72037 (16)	0.0210 (4)	
H22A	0.3321	0.6039	0.6531	0.025*	
H22B	0.1901	0.5940	0.6859	0.025*	
C23A	0.2794 (2)	0.67306 (9)	0.78133 (17)	0.0222 (4)	
H23A	0.2092	0.6761	0.8347	0.027*	
H23B	0.3586	0.6776	0.8317	0.027*	
C24A	0.2696 (2)	0.73224 (9)	0.69198 (16)	0.0214 (4)	
H24A	0.1933	0.7253	0.6384	0.026*	
H24B	0.3425	0.7300	0.6418	0.026*	
C25A	0.26435 (19)	0.80430 (10)	0.74575 (17)	0.0216 (4)	
H25A	0.1900	0.8059	0.7956	0.026*	
C27A	0.2446 (2)	0.85875 (10)	0.64785 (18)	0.0258 (4)	
H27A	0.2381	0.9043	0.6848	0.039*	
H27B	0.1673	0.8487	0.5992	0.039*	
H27C	0.3158	0.8582	0.5968	0.039*	
C26A	0.3789 (3)	0.82162 (12)	0.8271 (2)	0.0439 (6)	
H26A	0.3855	0.7891	0.8944	0.066*	
H26B	0.3711	0.8686	0.8579	0.066*	
H26C	0.4541	0.8183	0.7818	0.066*	
C21A	0.2224 (2)	0.53188 (10)	0.89955 (17)	0.0247 (4)	
H21A	0.2101	0.5760	0.9400	0.037*	
H21B	0.2552	0.4978	0.9582	0.037*	
H21C	0.1422	0.5158	0.8625	0.037*	
C19A	0.48099 (17)	0.42163 (9)	0.88552 (15)	0.0189 (3)	
H19A	0.5205	0.3780	0.9108	0.028*	
H19B	0.4464	0.4440	0.9547	0.028*	
H19C	0.5436	0.4518	0.8525	0.028*	
C18A	0.51117 (19)	0.18030 (10)	0.92217 (16)	0.0225 (4)	
H18A	0.5518	0.1355	0.9358	0.034*	
H18B	0.4589	0.1907	0.9887	0.034*	
H18C	0.5753	0.2161	0.9171	0.034*	
C13B	0.19713 (17)	0.15525 (9)	0.27013 (14)	0.0162 (3)	
C12B	0.13964 (19)	0.19277 (9)	0.15798 (15)	0.0210 (4)	
H12C	0.1743	0.1730	0.0855	0.025*	
H12D	0.0478	0.1855	0.1518	0.025*	
C11B	0.1675 (2)	0.27040 (10)	0.16367 (16)	0.0221 (4)	
H11C	0.1266	0.2928	0.0918	0.026*	
H11D	0.2590	0.2774	0.1613	0.026*	
C9B	0.12240 (17)	0.30577 (9)	0.27648 (14)	0.0167 (3)	
H9BA	0.0291	0.3010	0.2718	0.020*	
C10B	0.15000 (17)	0.38455 (9)	0.28127 (15)	0.0181 (3)	
C1B	0.0650 (2)	0.42059 (10)	0.18217 (16)	0.0234 (4)	
H1BA	0.0961	0.4090	0.1031	0.028*	
H1BB	−0.0209	0.4017	0.1842	0.028*	
C2B	0.0583 (2)	0.49911 (11)	0.19319 (18)	0.0287 (4)	
H2BA	0.1419	0.5192	0.1823	0.034*	
H2BB	−0.0011	0.5176	0.1295	0.034*	
C3B	0.0153 (2)	0.51952 (10)	0.31560 (17)	0.0260 (4)	
H3BA	−0.0694	0.5007	0.3260	0.031*	
H3BB	0.0112	0.5703	0.3219	0.031*	
C4B	0.10817 (19)	0.49093 (10)	0.41310 (17)	0.0224 (4)	
H4BA	0.1915	0.5120	0.4051	0.027*	
H4BB	0.0799	0.5032	0.4930	0.027*	
C5B	0.11862 (17)	0.41337 (9)	0.40335 (16)	0.0186 (3)	
C6B	0.09770 (17)	0.37372 (9)	0.49722 (15)	0.0193 (3)	
H6BA	0.0767	0.3960	0.5689	0.023*	
C7B	0.10474 (18)	0.29677 (9)	0.49828 (15)	0.0188 (3)	
H7BA	0.0188	0.2777	0.4970	0.023*	
H7BB	0.1503	0.2815	0.5736	0.023*	
C8B	0.17069 (17)	0.26817 (9)	0.39177 (15)	0.0166 (3)	
H8BA	0.2630	0.2757	0.4052	0.020*	
C14B	0.14447 (16)	0.19129 (9)	0.37893 (14)	0.0158 (3)	
H14B	0.0513	0.1869	0.3674	0.019*	
C15B	0.18369 (19)	0.14450 (9)	0.48515 (16)	0.0207 (3)	
H15C	0.1245	0.1486	0.5494	0.025*	
H15D	0.2691	0.1560	0.5186	0.025*	
C16B	0.17979 (19)	0.07137 (9)	0.43025 (15)	0.0211 (4)	
H16C	0.1140	0.0433	0.4654	0.025*	
H16D	0.2615	0.0480	0.4459	0.025*	
C17B	0.15011 (17)	0.08067 (9)	0.29291 (15)	0.0165 (3)	
H17B	0.0568	0.0818	0.2798	0.020*	
C20B	0.19557 (18)	0.01912 (9)	0.22096 (16)	0.0193 (3)	
H20B	0.2880	0.0145	0.2388	0.023*	
C22B	0.13311 (19)	−0.04703 (10)	0.26282 (16)	0.0211 (3)	
H22C	0.1581	−0.0539	0.3488	0.025*	
H22D	0.0412	−0.0401	0.2560	0.025*	
C23B	0.16278 (19)	−0.11291 (10)	0.19590 (17)	0.0224 (4)	
H23C	0.2543	−0.1157	0.1880	0.027*	
H23D	0.1213	−0.1111	0.1142	0.027*	
C24B	0.1194 (2)	−0.17724 (10)	0.25980 (18)	0.0244 (4)	
H24C	0.0269	−0.1762	0.2594	0.029*	
H24D	0.1525	−0.1752	0.3445	0.029*	
C25B	0.1584 (2)	−0.24600 (11)	0.2072 (2)	0.0298 (4)	
H25B	0.2517	−0.2456	0.2044	0.036*	
C26B	0.1032 (3)	−0.25629 (15)	0.0798 (2)	0.0513 (7)	
H26D	0.1277	−0.3016	0.0506	0.077*	
H26E	0.1346	−0.2203	0.0279	0.077*	
H26F	0.0116	−0.2535	0.0787	0.077*	
C27B	0.1243 (3)	−0.30568 (13)	0.2862 (3)	0.0440 (6)	
H27D	0.1571	−0.3486	0.2544	0.066*	
H27E	0.0329	−0.3088	0.2876	0.066*	
H27F	0.1608	−0.2982	0.3679	0.066*	
C21B	0.1720 (2)	0.02906 (10)	0.08577 (17)	0.0279 (4)	
H21D	0.1958	−0.0129	0.0441	0.042*	
H21E	0.2221	0.0679	0.0596	0.042*	
H21F	0.0829	0.0386	0.0669	0.042*	
C19B	0.34079 (18)	0.15689 (10)	0.27321 (18)	0.0236 (4)	
H19D	0.3696	0.2048	0.2752	0.035*	
H19E	0.3691	0.1341	0.2015	0.035*	
H19F	0.3754	0.1328	0.3449	0.035*	
C18B	0.28927 (19)	0.40012 (11)	0.2626 (2)	0.0274 (4)	
H18D	0.3060	0.4491	0.2776	0.041*	
H18E	0.3074	0.3888	0.1800	0.041*	
H18F	0.3427	0.3723	0.3183	0.041*	

### Atomic displacement parameters (Å^2^)

**Table:**

	*U*^11^	*U*^22^	*U*^33^	*U*^12^	*U*^13^	*U*^23^
C13A	0.0178 (8)	0.0130 (7)	0.0160 (7)	0.0006 (6)	0.0018 (6)	0.0005 (5)
C12A	0.0206 (8)	0.0152 (8)	0.0213 (8)	0.0003 (7)	0.0057 (6)	−0.0003 (6)
C11A	0.0247 (9)	0.0128 (8)	0.0201 (7)	0.0001 (7)	0.0085 (6)	0.0013 (6)
C9A	0.0184 (8)	0.0143 (7)	0.0155 (7)	0.0005 (6)	0.0031 (6)	0.0017 (6)
C10A	0.0199 (8)	0.0147 (8)	0.0138 (7)	0.0010 (6)	0.0015 (6)	0.0009 (6)
C1A	0.0242 (9)	0.0152 (8)	0.0189 (7)	0.0007 (7)	0.0038 (6)	0.0030 (6)
C2A	0.0303 (10)	0.0169 (8)	0.0231 (8)	−0.0021 (7)	0.0052 (7)	0.0026 (6)
C3A	0.0354 (11)	0.0142 (8)	0.0305 (9)	0.0001 (8)	0.0068 (8)	−0.0016 (7)
C4A	0.0319 (10)	0.0171 (9)	0.0261 (9)	0.0050 (8)	0.0087 (7)	0.0003 (7)
C5A	0.0208 (8)	0.0152 (8)	0.0197 (7)	0.0021 (7)	0.0020 (6)	−0.0006 (6)
C6A	0.0234 (9)	0.0171 (8)	0.0197 (8)	0.0013 (7)	0.0066 (6)	−0.0020 (6)
C7A	0.0247 (9)	0.0151 (8)	0.0192 (7)	0.0017 (7)	0.0062 (6)	0.0006 (6)
C8A	0.0182 (8)	0.0163 (7)	0.0151 (7)	−0.0005 (6)	0.0037 (6)	0.0008 (6)
C14A	0.0184 (8)	0.0134 (7)	0.0155 (7)	0.0003 (6)	0.0023 (6)	0.0016 (5)
C15A	0.0243 (9)	0.0152 (8)	0.0195 (7)	−0.0006 (7)	0.0059 (6)	0.0021 (6)
C16A	0.0254 (9)	0.0152 (8)	0.0193 (7)	−0.0008 (7)	0.0040 (6)	0.0024 (6)
C17A	0.0183 (8)	0.0135 (7)	0.0167 (7)	0.0001 (6)	0.0007 (6)	0.0006 (5)
C20A	0.0207 (8)	0.0141 (8)	0.0192 (7)	−0.0002 (7)	0.0018 (6)	−0.0003 (6)
C22A	0.0261 (10)	0.0154 (8)	0.0212 (8)	0.0006 (7)	0.0000 (7)	0.0001 (6)
C23A	0.0302 (10)	0.0139 (8)	0.0225 (8)	0.0017 (7)	0.0016 (7)	−0.0004 (6)
C24A	0.0278 (9)	0.0146 (8)	0.0217 (8)	0.0028 (7)	0.0002 (7)	−0.0007 (6)
C25A	0.0259 (9)	0.0146 (8)	0.0239 (8)	−0.0003 (7)	0.0003 (7)	0.0015 (6)
C27A	0.0295 (10)	0.0182 (9)	0.0293 (9)	0.0025 (8)	−0.0006 (8)	0.0031 (7)
C26A	0.0553 (16)	0.0225 (10)	0.0501 (14)	−0.0084 (11)	−0.0270 (12)	0.0039 (10)
C21A	0.0315 (10)	0.0167 (9)	0.0270 (9)	0.0017 (8)	0.0094 (8)	−0.0016 (7)
C19A	0.0210 (8)	0.0166 (8)	0.0189 (7)	0.0015 (7)	0.0007 (6)	0.0000 (6)
C18A	0.0287 (9)	0.0204 (9)	0.0177 (7)	0.0022 (7)	−0.0032 (7)	0.0011 (6)
C13B	0.0197 (8)	0.0128 (7)	0.0164 (7)	0.0003 (6)	0.0027 (6)	0.0008 (6)
C12B	0.0319 (10)	0.0163 (8)	0.0149 (7)	0.0013 (8)	0.0017 (6)	−0.0010 (6)
C11B	0.0331 (10)	0.0165 (8)	0.0171 (7)	0.0026 (7)	0.0059 (7)	0.0008 (6)
C9B	0.0197 (8)	0.0150 (8)	0.0154 (7)	−0.0003 (6)	0.0015 (6)	−0.0001 (6)
C10B	0.0212 (8)	0.0134 (7)	0.0201 (7)	0.0002 (7)	0.0047 (6)	−0.0001 (6)
C1B	0.0343 (10)	0.0174 (8)	0.0185 (8)	0.0012 (8)	0.0014 (7)	0.0012 (6)
C2B	0.0439 (12)	0.0183 (9)	0.0243 (9)	0.0014 (9)	0.0054 (8)	0.0048 (7)
C3B	0.0340 (11)	0.0180 (9)	0.0261 (9)	0.0024 (8)	0.0036 (8)	0.0013 (7)
C4B	0.0267 (9)	0.0164 (8)	0.0239 (8)	−0.0003 (7)	0.0018 (7)	−0.0031 (6)
C5B	0.0167 (8)	0.0167 (8)	0.0221 (8)	−0.0006 (7)	−0.0005 (6)	−0.0027 (6)
C6B	0.0207 (8)	0.0192 (9)	0.0176 (7)	−0.0005 (7)	−0.0013 (6)	−0.0036 (6)
C7B	0.0234 (9)	0.0180 (8)	0.0151 (7)	0.0014 (7)	0.0018 (6)	0.0002 (6)
C8B	0.0177 (8)	0.0148 (8)	0.0170 (7)	0.0014 (6)	0.0002 (6)	−0.0008 (6)
C14B	0.0173 (8)	0.0156 (8)	0.0146 (7)	−0.0006 (6)	0.0009 (6)	0.0014 (6)
C15B	0.0282 (9)	0.0163 (8)	0.0174 (7)	0.0014 (7)	−0.0009 (6)	0.0013 (6)
C16B	0.0289 (9)	0.0167 (8)	0.0174 (7)	−0.0002 (7)	0.0004 (7)	0.0017 (6)
C17B	0.0176 (8)	0.0147 (7)	0.0175 (7)	0.0000 (6)	0.0026 (6)	0.0001 (6)
C20B	0.0228 (9)	0.0147 (8)	0.0209 (8)	−0.0004 (7)	0.0045 (7)	−0.0006 (6)
C22B	0.0248 (9)	0.0168 (8)	0.0221 (8)	−0.0008 (7)	0.0052 (7)	−0.0012 (6)
C23B	0.0253 (9)	0.0169 (8)	0.0255 (8)	−0.0019 (7)	0.0057 (7)	−0.0036 (7)
C24B	0.0301 (10)	0.0163 (8)	0.0275 (9)	−0.0010 (8)	0.0071 (8)	−0.0033 (7)
C25B	0.0273 (10)	0.0216 (10)	0.0411 (11)	−0.0025 (8)	0.0072 (9)	−0.0068 (8)
C26B	0.074 (2)	0.0364 (14)	0.0431 (13)	−0.0008 (14)	−0.0009 (13)	−0.0185 (11)
C27B	0.0476 (15)	0.0199 (11)	0.0666 (17)	−0.0006 (11)	0.0206 (13)	0.0001 (11)
C21B	0.0455 (12)	0.0188 (9)	0.0205 (8)	−0.0020 (9)	0.0100 (8)	−0.0003 (7)
C19B	0.0233 (9)	0.0170 (8)	0.0312 (9)	−0.0001 (7)	0.0075 (7)	−0.0013 (7)
C18B	0.0265 (10)	0.0198 (9)	0.0372 (10)	−0.0044 (8)	0.0117 (8)	−0.0027 (8)

### Geometric parameters (Å, °)

**Table:**

C13A—C19A	1.539 (2)	C13B—C19B	1.535 (3)
C13A—C12A	1.539 (2)	C13B—C14B	1.540 (2)
C13A—C14A	1.547 (2)	C13B—C12B	1.543 (2)
C13A—C17A	1.560 (2)	C13B—C17B	1.558 (2)
C12A—C11A	1.541 (2)	C12B—C11B	1.536 (3)
C12A—H12A	0.9900	C12B—H12C	0.9900
C12A—H12B	0.9900	C12B—H12D	0.9900
C11A—C9A	1.546 (2)	C11B—C9B	1.540 (2)
C11A—H11A	0.9900	C11B—H11C	0.9900
C11A—H11B	0.9900	C11B—H11D	0.9900
C9A—C8A	1.541 (2)	C9B—C8B	1.541 (2)
C9A—C10A	1.561 (2)	C9B—C10B	1.557 (2)
C9A—H9AA	1.0000	C9B—H9BA	1.0000
C10A—C5A	1.532 (2)	C10B—C5B	1.531 (2)
C10A—C18A	1.544 (2)	C10B—C18B	1.547 (3)
C10A—C1A	1.548 (3)	C10B—C1B	1.554 (3)
C1A—C2A	1.532 (3)	C1B—C2B	1.531 (3)
C1A—H1AA	0.9900	C1B—H1BA	0.9900
C1A—H1AB	0.9900	C1B—H1BB	0.9900
C2A—C3A	1.526 (3)	C2B—C3B	1.523 (3)
C2A—H2AA	0.9900	C2B—H2BA	0.9900
C2A—H2AB	0.9900	C2B—H2BB	0.9900
C3A—C4A	1.521 (3)	C3B—C4B	1.531 (3)
C3A—H3AA	0.9900	C3B—H3BA	0.9900
C3A—H3AB	0.9900	C3B—H3BB	0.9900
C4A—C5A	1.510 (3)	C4B—C5B	1.514 (3)
C4A—H4AA	0.9900	C4B—H4BA	0.9900
C4A—H4AB	0.9900	C4B—H4BB	0.9900
C5A—C6A	1.331 (2)	C5B—C6B	1.331 (3)
C6A—C7A	1.494 (3)	C6B—C7B	1.495 (3)
C6A—H6AA	0.9500	C6B—H6BA	0.9500
C7A—C8A	1.533 (2)	C7B—C8B	1.526 (2)
C7A—H7AA	0.9900	C7B—H7BA	0.9900
C7A—H7AB	0.9900	C7B—H7BB	0.9900
C8A—C14A	1.525 (2)	C8B—C14B	1.523 (2)
C8A—H8AA	1.0000	C8B—H8BA	1.0000
C14A—C15A	1.526 (2)	C14B—C15B	1.532 (2)
C14A—H14A	1.0000	C14B—H14B	1.0000
C15A—C16A	1.545 (3)	C15B—C16B	1.546 (3)
C15A—H15A	0.9900	C15B—H15C	0.9900
C15A—H15B	0.9900	C15B—H15D	0.9900
C16A—C17A	1.565 (3)	C16B—C17B	1.558 (2)
C16A—H16A	0.9900	C16B—H16C	0.9900
C16A—H16B	0.9900	C16B—H16D	0.9900
C17A—C20A	1.544 (2)	C17B—C20B	1.536 (2)
C17A—H17A	1.0000	C17B—H17B	1.0000
C20A—C21A	1.534 (3)	C20B—C21B	1.528 (3)
C20A—C22A	1.542 (3)	C20B—C22B	1.534 (3)
C20A—H20A	1.0000	C20B—H20B	1.0000
C22A—C23A	1.528 (3)	C22B—C23B	1.525 (3)
C22A—H22A	0.9900	C22B—H22C	0.9900
C22A—H22B	0.9900	C22B—H22D	0.9900
C23A—C24A	1.521 (3)	C23B—C24B	1.525 (3)
C23A—H23A	0.9900	C23B—H23C	0.9900
C23A—H23B	0.9900	C23B—H23D	0.9900
C24A—C25A	1.525 (3)	C24B—C25B	1.527 (3)
C24A—H24A	0.9900	C24B—H24C	0.9900
C24A—H24B	0.9900	C24B—H24D	0.9900
C25A—C26A	1.516 (3)	C25B—C27B	1.515 (3)
C25A—C27A	1.526 (3)	C25B—C26B	1.519 (3)
C25A—H25A	1.0000	C25B—H25B	1.0000
C27A—H27A	0.9800	C26B—H26D	0.9800
C27A—H27B	0.9800	C26B—H26E	0.9800
C27A—H27C	0.9800	C26B—H26F	0.9800
C26A—H26A	0.9800	C27B—H27D	0.9800
C26A—H26B	0.9800	C27B—H27E	0.9800
C26A—H26C	0.9800	C27B—H27F	0.9800
C21A—H21A	0.9800	C21B—H21D	0.9800
C21A—H21B	0.9800	C21B—H21E	0.9800
C21A—H21C	0.9800	C21B—H21F	0.9800
C19A—H19A	0.9800	C19B—H19D	0.9800
C19A—H19B	0.9800	C19B—H19E	0.9800
C19A—H19C	0.9800	C19B—H19F	0.9800
C18A—H18A	0.9800	C18B—H18D	0.9800
C18A—H18B	0.9800	C18B—H18E	0.9800
C18A—H18C	0.9800	C18B—H18F	0.9800
			
C19A—C13A—C12A	110.89 (14)	C19B—C13B—C14B	112.88 (14)
C19A—C13A—C14A	112.52 (14)	C19B—C13B—C12B	110.78 (15)
C12A—C13A—C14A	105.78 (14)	C14B—C13B—C12B	106.28 (14)
C19A—C13A—C17A	110.17 (14)	C19B—C13B—C17B	110.39 (14)
C12A—C13A—C17A	116.84 (14)	C14B—C13B—C17B	98.90 (13)
C14A—C13A—C17A	100.15 (13)	C12B—C13B—C17B	117.06 (14)
C13A—C12A—C11A	111.99 (15)	C11B—C12B—C13B	111.28 (15)
C13A—C12A—H12A	109.2	C11B—C12B—H12C	109.4
C11A—C12A—H12A	109.2	C13B—C12B—H12C	109.4
C13A—C12A—H12B	109.2	C11B—C12B—H12D	109.4
C11A—C12A—H12B	109.2	C13B—C12B—H12D	109.4
H12A—C12A—H12B	107.9	H12C—C12B—H12D	108.0
C12A—C11A—C9A	114.25 (14)	C12B—C11B—C9B	113.63 (15)
C12A—C11A—H11A	108.7	C12B—C11B—H11C	108.8
C9A—C11A—H11A	108.7	C9B—C11B—H11C	108.8
C12A—C11A—H11B	108.7	C12B—C11B—H11D	108.8
C9A—C11A—H11B	108.7	C9B—C11B—H11D	108.8
H11A—C11A—H11B	107.6	H11C—C11B—H11D	107.7
C8A—C9A—C11A	111.12 (14)	C11B—C9B—C8B	111.58 (14)
C8A—C9A—C10A	112.95 (14)	C11B—C9B—C10B	113.40 (14)
C11A—C9A—C10A	113.56 (13)	C8B—C9B—C10B	112.55 (14)
C8A—C9A—H9AA	106.2	C11B—C9B—H9BA	106.2
C11A—C9A—H9AA	106.2	C8B—C9B—H9BA	106.2
C10A—C9A—H9AA	106.2	C10B—C9B—H9BA	106.2
C5A—C10A—C18A	108.67 (15)	C5B—C10B—C18B	108.45 (15)
C5A—C10A—C1A	108.01 (14)	C5B—C10B—C1B	108.60 (15)
C18A—C10A—C1A	109.59 (14)	C18B—C10B—C1B	109.91 (16)
C5A—C10A—C9A	110.26 (13)	C5B—C10B—C9B	109.71 (14)
C18A—C10A—C9A	111.26 (14)	C18B—C10B—C9B	111.67 (15)
C1A—C10A—C9A	108.99 (14)	C1B—C10B—C9B	108.45 (14)
C2A—C1A—C10A	114.46 (15)	C2B—C1B—C10B	114.78 (16)
C2A—C1A—H1AA	108.6	C2B—C1B—H1BA	108.6
C10A—C1A—H1AA	108.6	C10B—C1B—H1BA	108.6
C2A—C1A—H1AB	108.6	C2B—C1B—H1BB	108.6
C10A—C1A—H1AB	108.6	C10B—C1B—H1BB	108.6
H1AA—C1A—H1AB	107.6	H1BA—C1B—H1BB	107.5
C3A—C2A—C1A	110.96 (15)	C3B—C2B—C1B	110.46 (16)
C3A—C2A—H2AA	109.4	C3B—C2B—H2BA	109.6
C1A—C2A—H2AA	109.4	C1B—C2B—H2BA	109.6
C3A—C2A—H2AB	109.4	C3B—C2B—H2BB	109.6
C1A—C2A—H2AB	109.4	C1B—C2B—H2BB	109.6
H2AA—C2A—H2AB	108.0	H2BA—C2B—H2BB	108.1
C4A—C3A—C2A	110.48 (16)	C2B—C3B—C4B	109.01 (17)
C4A—C3A—H3AA	109.6	C2B—C3B—H3BA	109.9
C2A—C3A—H3AA	109.6	C4B—C3B—H3BA	109.9
C4A—C3A—H3AB	109.6	C2B—C3B—H3BB	109.9
C2A—C3A—H3AB	109.6	C4B—C3B—H3BB	109.9
H3AA—C3A—H3AB	108.1	H3BA—C3B—H3BB	108.3
C5A—C4A—C3A	112.29 (17)	C5B—C4B—C3B	110.89 (16)
C5A—C4A—H4AA	109.1	C5B—C4B—H4BA	109.5
C3A—C4A—H4AA	109.1	C3B—C4B—H4BA	109.5
C5A—C4A—H4AB	109.1	C5B—C4B—H4BB	109.5
C3A—C4A—H4AB	109.1	C3B—C4B—H4BB	109.5
H4AA—C4A—H4AB	107.9	H4BA—C4B—H4BB	108.0
C6A—C5A—C4A	120.28 (17)	C6B—C5B—C4B	120.07 (17)
C6A—C5A—C10A	123.66 (16)	C6B—C5B—C10B	123.25 (16)
C4A—C5A—C10A	116.05 (15)	C4B—C5B—C10B	116.66 (15)
C5A—C6A—C7A	124.77 (16)	C5B—C6B—C7B	124.93 (17)
C5A—C6A—H6AA	117.6	C5B—C6B—H6BA	117.5
C7A—C6A—H6AA	117.6	C7B—C6B—H6BA	117.5
C6A—C7A—C8A	112.76 (14)	C6B—C7B—C8B	112.48 (15)
C6A—C7A—H7AA	109.0	C6B—C7B—H7BA	109.1
C8A—C7A—H7AA	109.0	C8B—C7B—H7BA	109.1
C6A—C7A—H7AB	109.0	C6B—C7B—H7BB	109.1
C8A—C7A—H7AB	109.0	C8B—C7B—H7BB	109.1
H7AA—C7A—H7AB	107.8	H7BA—C7B—H7BB	107.8
C14A—C8A—C7A	110.30 (14)	C14B—C8B—C7B	109.77 (14)
C14A—C8A—C9A	109.76 (14)	C14B—C8B—C9B	109.59 (13)
C7A—C8A—C9A	110.29 (14)	C7B—C8B—C9B	109.46 (14)
C14A—C8A—H8AA	108.8	C14B—C8B—H8BA	109.3
C7A—C8A—H8AA	108.8	C7B—C8B—H8BA	109.3
C9A—C8A—H8AA	108.8	C9B—C8B—H8BA	109.3
C8A—C14A—C15A	118.18 (15)	C8B—C14B—C15B	117.91 (14)
C8A—C14A—C13A	114.77 (13)	C8B—C14B—C13B	116.41 (14)
C15A—C14A—C13A	104.79 (14)	C15B—C14B—C13B	104.30 (14)
C8A—C14A—H14A	106.1	C8B—C14B—H14B	105.7
C15A—C14A—H14A	106.1	C15B—C14B—H14B	105.7
C13A—C14A—H14A	106.1	C13B—C14B—H14B	105.7
C14A—C15A—C16A	103.38 (14)	C14B—C15B—C16B	103.74 (14)
C14A—C15A—H15A	111.1	C14B—C15B—H15C	111.0
C16A—C15A—H15A	111.1	C16B—C15B—H15C	111.0
C14A—C15A—H15B	111.1	C14B—C15B—H15D	111.0
C16A—C15A—H15B	111.1	C16B—C15B—H15D	111.0
H15A—C15A—H15B	109.1	H15C—C15B—H15D	109.0
C15A—C16A—C17A	107.32 (14)	C15B—C16B—C17B	106.46 (14)
C15A—C16A—H16A	110.3	C15B—C16B—H16C	110.4
C17A—C16A—H16A	110.3	C17B—C16B—H16C	110.4
C15A—C16A—H16B	110.3	C15B—C16B—H16D	110.4
C17A—C16A—H16B	110.3	C17B—C16B—H16D	110.4
H16A—C16A—H16B	108.5	H16C—C16B—H16D	108.6
C20A—C17A—C13A	119.47 (13)	C20B—C17B—C16B	111.98 (14)
C20A—C17A—C16A	111.09 (14)	C20B—C17B—C13B	121.36 (14)
C13A—C17A—C16A	103.80 (14)	C16B—C17B—C13B	102.77 (13)
C20A—C17A—H17A	107.3	C20B—C17B—H17B	106.6
C13A—C17A—H17A	107.3	C16B—C17B—H17B	106.6
C16A—C17A—H17A	107.3	C13B—C17B—H17B	106.6
C21A—C20A—C22A	110.25 (16)	C21B—C20B—C22B	111.07 (16)
C21A—C20A—C17A	113.12 (15)	C21B—C20B—C17B	112.43 (15)
C22A—C20A—C17A	109.77 (14)	C22B—C20B—C17B	109.43 (14)
C21A—C20A—H20A	107.8	C21B—C20B—H20B	107.9
C22A—C20A—H20A	107.8	C22B—C20B—H20B	107.9
C17A—C20A—H20A	107.8	C17B—C20B—H20B	107.9
C23A—C22A—C20A	115.18 (15)	C23B—C22B—C20B	116.35 (15)
C23A—C22A—H22A	108.5	C23B—C22B—H22C	108.2
C20A—C22A—H22A	108.5	C20B—C22B—H22C	108.2
C23A—C22A—H22B	108.5	C23B—C22B—H22D	108.2
C20A—C22A—H22B	108.5	C20B—C22B—H22D	108.2
H22A—C22A—H22B	107.5	H22C—C22B—H22D	107.4
C24A—C23A—C22A	112.64 (15)	C22B—C23B—C24B	112.19 (15)
C24A—C23A—H23A	109.1	C22B—C23B—H23C	109.2
C22A—C23A—H23A	109.1	C24B—C23B—H23C	109.2
C24A—C23A—H23B	109.1	C22B—C23B—H23D	109.2
C22A—C23A—H23B	109.1	C24B—C23B—H23D	109.2
H23A—C23A—H23B	107.8	H23C—C23B—H23D	107.9
C23A—C24A—C25A	115.89 (15)	C23B—C24B—C25B	115.87 (16)
C23A—C24A—H24A	108.3	C23B—C24B—H24C	108.3
C25A—C24A—H24A	108.3	C25B—C24B—H24C	108.3
C23A—C24A—H24B	108.3	C23B—C24B—H24D	108.3
C25A—C24A—H24B	108.3	C25B—C24B—H24D	108.3
H24A—C24A—H24B	107.4	H24C—C24B—H24D	107.4
C26A—C25A—C24A	113.04 (18)	C27B—C25B—C26B	110.6 (2)
C26A—C25A—C27A	110.26 (17)	C27B—C25B—C24B	111.18 (19)
C24A—C25A—C27A	111.07 (15)	C26B—C25B—C24B	112.1 (2)
C26A—C25A—H25A	107.4	C27B—C25B—H25B	107.6
C24A—C25A—H25A	107.4	C26B—C25B—H25B	107.6
C27A—C25A—H25A	107.4	C24B—C25B—H25B	107.6
C25A—C27A—H27A	109.5	C25B—C26B—H26D	109.5
C25A—C27A—H27B	109.5	C25B—C26B—H26E	109.5
H27A—C27A—H27B	109.5	H26D—C26B—H26E	109.5
C25A—C27A—H27C	109.5	C25B—C26B—H26F	109.5
H27A—C27A—H27C	109.5	H26D—C26B—H26F	109.5
H27B—C27A—H27C	109.5	H26E—C26B—H26F	109.5
C25A—C26A—H26A	109.5	C25B—C27B—H27D	109.5
C25A—C26A—H26B	109.5	C25B—C27B—H27E	109.5
H26A—C26A—H26B	109.5	H27D—C27B—H27E	109.5
C25A—C26A—H26C	109.5	C25B—C27B—H27F	109.5
H26A—C26A—H26C	109.5	H27D—C27B—H27F	109.5
H26B—C26A—H26C	109.5	H27E—C27B—H27F	109.5
C20A—C21A—H21A	109.5	C20B—C21B—H21D	109.5
C20A—C21A—H21B	109.5	C20B—C21B—H21E	109.5
H21A—C21A—H21B	109.5	H21D—C21B—H21E	109.5
C20A—C21A—H21C	109.5	C20B—C21B—H21F	109.5
H21A—C21A—H21C	109.5	H21D—C21B—H21F	109.5
H21B—C21A—H21C	109.5	H21E—C21B—H21F	109.5
C13A—C19A—H19A	109.5	C13B—C19B—H19D	109.5
C13A—C19A—H19B	109.5	C13B—C19B—H19E	109.5
H19A—C19A—H19B	109.5	H19D—C19B—H19E	109.5
C13A—C19A—H19C	109.5	C13B—C19B—H19F	109.5
H19A—C19A—H19C	109.5	H19D—C19B—H19F	109.5
H19B—C19A—H19C	109.5	H19E—C19B—H19F	109.5
C10A—C18A—H18A	109.5	C10B—C18B—H18D	109.5
C10A—C18A—H18B	109.5	C10B—C18B—H18E	109.5
H18A—C18A—H18B	109.5	H18D—C18B—H18E	109.5
C10A—C18A—H18C	109.5	C10B—C18B—H18F	109.5
H18A—C18A—H18C	109.5	H18D—C18B—H18F	109.5
H18B—C18A—H18C	109.5	H18E—C18B—H18F	109.5
			
C19A—C13A—C12A—C11A	−66.17 (19)	C19B—C13B—C12B—C11B	−66.9 (2)
C14A—C13A—C12A—C11A	56.10 (17)	C14B—C13B—C12B—C11B	56.03 (19)
C17A—C13A—C12A—C11A	166.48 (14)	C17B—C13B—C12B—C11B	165.30 (15)
C13A—C12A—C11A—C9A	−54.2 (2)	C13B—C12B—C11B—C9B	−56.5 (2)
C12A—C11A—C9A—C8A	49.8 (2)	C12B—C11B—C9B—C8B	52.1 (2)
C12A—C11A—C9A—C10A	178.40 (15)	C12B—C11B—C9B—C10B	−179.56 (16)
C8A—C9A—C10A—C5A	−41.89 (19)	C11B—C9B—C10B—C5B	−172.31 (15)
C11A—C9A—C10A—C5A	−169.59 (15)	C8B—C9B—C10B—C5B	−44.5 (2)
C8A—C9A—C10A—C18A	78.76 (17)	C11B—C9B—C10B—C18B	−52.0 (2)
C11A—C9A—C10A—C18A	−48.94 (19)	C8B—C9B—C10B—C18B	75.82 (19)
C8A—C9A—C10A—C1A	−160.29 (13)	C11B—C9B—C10B—C1B	69.22 (19)
C11A—C9A—C10A—C1A	72.01 (17)	C8B—C9B—C10B—C1B	−162.93 (15)
C5A—C10A—C1A—C2A	50.67 (18)	C5B—C10B—C1B—C2B	47.4 (2)
C18A—C10A—C1A—C2A	−67.56 (19)	C18B—C10B—C1B—C2B	−71.1 (2)
C9A—C10A—C1A—C2A	170.48 (14)	C9B—C10B—C1B—C2B	166.53 (16)
C10A—C1A—C2A—C3A	−55.9 (2)	C10B—C1B—C2B—C3B	−56.2 (2)
C1A—C2A—C3A—C4A	55.4 (2)	C1B—C2B—C3B—C4B	59.5 (2)
C2A—C3A—C4A—C5A	−53.9 (2)	C2B—C3B—C4B—C5B	−58.1 (2)
C3A—C4A—C5A—C6A	−126.64 (19)	C3B—C4B—C5B—C6B	−124.47 (19)
C3A—C4A—C5A—C10A	53.0 (2)	C3B—C4B—C5B—C10B	53.8 (2)
C18A—C10A—C5A—C6A	−110.9 (2)	C18B—C10B—C5B—C6B	−108.9 (2)
C1A—C10A—C5A—C6A	130.32 (18)	C1B—C10B—C5B—C6B	131.66 (19)
C9A—C10A—C5A—C6A	11.3 (2)	C9B—C10B—C5B—C6B	13.3 (2)
C18A—C10A—C5A—C4A	69.5 (2)	C18B—C10B—C5B—C4B	72.8 (2)
C1A—C10A—C5A—C4A	−49.3 (2)	C1B—C10B—C5B—C4B	−46.6 (2)
C9A—C10A—C5A—C4A	−168.31 (15)	C9B—C10B—C5B—C4B	−164.98 (15)
C4A—C5A—C6A—C7A	−177.59 (18)	C4B—C5B—C6B—C7B	179.78 (17)
C10A—C5A—C6A—C7A	2.8 (3)	C10B—C5B—C6B—C7B	1.6 (3)
C5A—C6A—C7A—C8A	13.8 (3)	C5B—C6B—C7B—C8B	14.7 (3)
C6A—C7A—C8A—C14A	−164.74 (15)	C6B—C7B—C8B—C14B	−164.85 (14)
C6A—C7A—C8A—C9A	−43.3 (2)	C6B—C7B—C8B—C9B	−44.5 (2)
C11A—C9A—C8A—C14A	−50.22 (18)	C11B—C9B—C8B—C14B	−49.27 (19)
C10A—C9A—C8A—C14A	−179.19 (13)	C10B—C9B—C8B—C14B	−178.07 (14)
C11A—C9A—C8A—C7A	−171.95 (14)	C11B—C9B—C8B—C7B	−169.70 (15)
C10A—C9A—C8A—C7A	59.08 (18)	C10B—C9B—C8B—C7B	61.50 (19)
C7A—C8A—C14A—C15A	−54.6 (2)	C7B—C8B—C14B—C15B	−58.9 (2)
C9A—C8A—C14A—C15A	−176.35 (14)	C9B—C8B—C14B—C15B	−179.11 (15)
C7A—C8A—C14A—C13A	−179.11 (15)	C7B—C8B—C14B—C13B	176.03 (14)
C9A—C8A—C14A—C13A	59.16 (18)	C9B—C8B—C14B—C13B	55.80 (19)
C19A—C13A—C14A—C8A	60.24 (19)	C19B—C13B—C14B—C8B	63.10 (19)
C12A—C13A—C14A—C8A	−60.98 (18)	C12B—C13B—C14B—C8B	−58.53 (19)
C17A—C13A—C14A—C8A	177.21 (14)	C17B—C13B—C14B—C8B	179.78 (14)
C19A—C13A—C14A—C15A	−71.04 (17)	C19B—C13B—C14B—C15B	−68.64 (18)
C12A—C13A—C14A—C15A	167.74 (14)	C12B—C13B—C14B—C15B	169.73 (15)
C17A—C13A—C14A—C15A	45.92 (16)	C17B—C13B—C14B—C15B	48.03 (16)
C8A—C14A—C15A—C16A	−165.17 (14)	C8B—C14B—C15B—C16B	−164.14 (15)
C13A—C14A—C15A—C16A	−35.89 (17)	C13B—C14B—C15B—C16B	−33.27 (18)
C14A—C15A—C16A—C17A	11.55 (18)	C14B—C15B—C16B—C17B	4.96 (19)
C19A—C13A—C17A—C20A	−42.9 (2)	C15B—C16B—C17B—C20B	156.30 (15)
C12A—C13A—C17A—C20A	84.8 (2)	C15B—C16B—C17B—C13B	24.47 (18)
C14A—C13A—C17A—C20A	−161.57 (15)	C19B—C13B—C17B—C20B	−51.0 (2)
C19A—C13A—C17A—C16A	81.50 (16)	C14B—C13B—C17B—C20B	−169.59 (15)
C12A—C13A—C17A—C16A	−150.80 (15)	C12B—C13B—C17B—C20B	76.9 (2)
C14A—C13A—C17A—C16A	−37.20 (16)	C19B—C13B—C17B—C16B	74.95 (17)
C15A—C16A—C17A—C20A	146.07 (15)	C14B—C13B—C17B—C16B	−43.61 (16)
C15A—C16A—C17A—C13A	16.45 (18)	C12B—C13B—C17B—C16B	−157.09 (15)
C13A—C17A—C20A—C21A	−57.5 (2)	C16B—C17B—C20B—C21B	−177.56 (16)
C16A—C17A—C20A—C21A	−178.32 (15)	C13B—C17B—C20B—C21B	−55.9 (2)
C13A—C17A—C20A—C22A	178.86 (15)	C16B—C17B—C20B—C22B	58.5 (2)
C16A—C17A—C20A—C22A	58.08 (19)	C13B—C17B—C20B—C22B	−179.80 (15)
C21A—C20A—C22A—C23A	65.1 (2)	C21B—C20B—C22B—C23B	52.2 (2)
C17A—C20A—C22A—C23A	−169.64 (16)	C17B—C20B—C22B—C23B	176.93 (16)
C20A—C22A—C23A—C24A	166.49 (17)	C20B—C22B—C23B—C24B	167.99 (17)
C22A—C23A—C24A—C25A	176.60 (17)	C22B—C23B—C24B—C25B	−173.25 (18)
C23A—C24A—C25A—C26A	59.8 (2)	C23B—C24B—C25B—C27B	173.0 (2)
C23A—C24A—C25A—C27A	−175.69 (18)	C23B—C24B—C25B—C26B	−62.6 (3)
